# Nasopharyngeal microbiota as a diagnostic biomarker for chronic rhinosinusitis with nasal polyps

**DOI:** 10.3389/fcimb.2026.1865473

**Published:** 2026-10-13

**Authors:** Xiao Liu, Wenfei Yang, Yingbin Wang, Shaoping Peng, Jing Zhou, Quanpeng Qiu

**Affiliations:** 1 Department of Otolaryngology-Head and Neck Surgery, First Affiliated Hospital of Gannan Medical University, Ganzhou, Jiangxi, China; 2 Clinical Research Center, First Affiliated Hospital of Gannan Medical University, Ganzhou, Jiangxi, China

**Keywords:** biomarkers, chronic rhinosinusitis with nasal polyps, diagnostic model, nasopharyngeal microbiota, recurrence prediction

## Abstract

**Background:**

Chronic rhinosinusitis with nasal polyps (CRSwNP) is a common inflammatory disorder with high postoperative recurrence rates. The nasopharyngeal microbiome may play a role in disease pathogenesis, but its diagnostic and prognostic utility remains unclear. This study characterized the nasopharyngeal microbiota in CRSwNP patients and evaluated its potential as a biomarker.

**Methods:**

32 nasopharyngeal swab samples from CRSwNP patients and 11 from controls were collected and subjected to 16S rRNA gene sequencing. Microbial diversity, species composition, and differential abundance were analyzed. A diagnostic model was constructed using LASSO regression, with feature selection performed entirely within a 10-fold cross-validation framework, and model stability was validated via 100 Bootstrap resampling iterations. For recurrence prediction, due to the limited number of events (n=10), only exploratory univariate screening was conducted, and the stability of candidate biomarkers was evaluated using Bootstrap validation with internal feature selection (300 iterations).

**Results:**

CRSwNP patients exhibited significantly altered microbial composition at phylum and genus levels, as well as higher Shannon and Chao1 indices compared with controls, although the clinical and biological significance of this diversity increase requires further investigation. The three-genus combined diagnostic panel consisting of *Fibrobacter*, *Ruminococcaceae_UCG-002*, and *Staphylococcus* demonstrated favorable diagnostic performance. For recurrence prediction, *Helicobacter* and *Parabacteroides* were identified as stable candidate biomarkers, with Bootstrap selection frequencies of 24.0% and 27.3%, respectively. The recurrence-related findings are exploratory hypothesis-generating results and do not possess clinical predictive capability; they require validation in an independent large-cohort study.

**Conclusions:**

The nasopharyngeal microbiota of CRSwNP patients exhibited significant alterations. The three-genus combined diagnostic panel demonstrated good discriminatory ability, supporting its potential as a non-invasive diagnostic biomarker. The identification of recurrence-related markers provides candidate targets for future research; however, due to the limited sample size, they currently lack clinical predictive capability. This study provides foundational data for microbiome research and clinical translation in CRSwNP.

## Introduction

Chronic Rhinosinusitis (CRS) is a common chronic inflammatory disease of the upper airway (Hellings et al., 2024). It significantly impairs patients’ quality of life and imposes a substantial socioeconomic burden. Based on the presence or absence of nasal polyps, CRS can be classified into two clinical phenotypes: Chronic Rhinosinusitis with Nasal Polyps (CRSwNP) and Chronic Rhinosinusitis without Nasal Polyps (Hellings et al., 2024). Among these, CRSwNP is characterized by a more pronounced Th2-skewed inflammatory profile, marked by eosinophilic infiltration, elevated levels of cytokines such as IL-5 and IL-13, and associated tissue remodeling and polyp formation (Cheng et al., 2025; Zhao et al., 2025; Li et al., 2026). Although functional endoscopic sinus surgery is one of the primary treatment modalities for CRSwNP (Cheng et al., 2025), the postoperative recurrence rate remains high, and reaching even higher levels in patients with refractory disease (Dorismond et al., 2025). Recurrent nasal polyps not only necessitate multiple surgical interventions but also significantly compromise olfactory function and quality of life (Huber et al., 2024; Seys et al., 2025). Therefore, exploring the pathogenesis of CRSwNP and identifying reliable diagnostic and prognostic biomarkers is of significant clinical importance.

Advances in high-throughput sequencing technologies have garnered increasing attention to the role of the human microbiome in chronic inflammatory diseases. The upper respiratory tract is not a sterile environment but is colonized by complex and diverse microbial communities, which play critical roles in maintaining mucosal immune homeostasis, defending against pathogen invasion, and regulating local inflammatory responses (Huang et al., 2025; Ji et al., 2026). Studies have shown that the microbial composition of the nasal and sinus mucosa in patients with CRS differs significantly from that of healthy individuals, primarily manifesting as reduced microbial diversity and overgrowth of specific bacterial genera (Hoggard et al., 2017).

Although previous studies have documented alterations in the nasal microbiota of patients with CRS, several key scientific questions remain unresolved. The compositional characteristics of the nasopharyngeal microbiota in patients with CRSwNP and their association with disease severity remain poorly defined. Systematic evaluations regarding the potential of microbial markers as non-invasive diagnostic tools for CRSwNP, including their diagnostic performance, are currently lacking. Postoperative recurrence represents a major challenge in the clinical management of CRSwNP, yet reliable biomarkers capable of effectively predicting individual recurrence risk are currently unavailable. Should microbial markers associated with recurrence be identified from the nasopharyngeal microbiota, they would facilitate preoperative risk stratification, guide postoperative follow-up strategies, and support individualized treatment decisions (Liang et al., 2026).

Building upon this research background, this study aims to compare the compositional differences of nasopharyngeal microbiota between CRSwNP patients and controls, identify CRSwNP-associated microbial features, construct a diagnostic model for CRSwNP based on nasopharyngeal microbial biomarkers and evaluate its diagnostic performance, and analyze the correlations between microbial biomarkers and disease severity indicators to explore their clinical value in disease assessment. Regarding recurrence, due to sample size limitations, only exploratory univariate screening was performed to identify candidate biomarkers. Through this study, we expect to provide novel microbial biomarkers for non-invasive diagnosis of CRSwNP and offer candidate targets for future recurrence prediction research.

## Materials and methods

### Subjects and sample collection

This study enrolled patients with chronic rhinosinusitis with nasal polyps (CRSwNP) and those with nasal septum deviation (control group) treated at the Department of Otolaryngology-Head and Neck Surgery of the First Affiliated Hospital of Gannan Medical University. The diagnosis of CRS was based on the criteria outlined in the Chinese Guidelines for Diagnosis and Treatment of Chronic Rhinosinusitis and was confirmed by nasal endoscopy and sinus computed tomography (CT) demonstrating nasal polyps. Patients with nasal septum deviation had no history of chronic rhinosinusitis and exhibited no evidence of nasal mucosal inflammation on endoscopic examination. Exclusion criteria were as follows: (1) allergic fungal rhinosinusitis; (2) antrochoanal polyp; (3) cystic fibrosis; (4) primary ciliary dyskinesia; (5) immunodeficiency; (6) sinonasal malignancy; (7) recent systemic corticosteroid use (within 4 weeks prior to sampling); (8) recent antibiotic use (within 2 weeks prior to sampling); (9) pregnancy or lactation; (10) incomplete clinical data.

Nasopharyngeal swab samples were collected from all subjects under endoscopic guidance. A sterile, flexible flocked swab was gently inserted through the nasal cavity along the floor of the nasal passage until resistance was felt at the posterior nasopharyngeal wall. The swab was then rotated for approximately 5 seconds to absorb mucosal secretions from the nasopharyngeal mucosa, carefully withdrawn without touching the nasal turbinates or oral cavity, and immediately placed into a sterile tube containing transport medium. All samples were stored at −80 °C until DNA extraction. Clinical data were also recorded for all subjects, including age, sex, peripheral blood eosinophil ratio, Lund–Mackay score, Lund–Kennedy score, VAS score, and comorbidities such as asthma and rhinitis. This study was approved by the First Affiliated Hospital of Gannan Medical University’s Ethics Committee (Ethics Lot Number: LLSC-2022110901), and all methods were performed in accordance with the relevant guidelines.

### Postoperative follow-up and definition of recurrence

All patients with CRS underwent functional endoscopic sinus surgery and were regularly followed up for at least 12 months postoperatively. Recurrence was defined as endoscopically visible polyp regrowth with a Lund–Kennedy score ≥2 at 12 months after surgery, accompanied by the return of symptoms requiring medical or surgical intervention. Based on the follow-up results, patients with CRSwNP were stratified into a non-recurrence group and a recurrence group.

### DNA extraction and 16S rRNA gene sequencing

Total DNA was extracted from nasopharyngeal swab samples. DNA concentration and purity were measured using a NanoDrop 2000 spectrophotometer, and DNA integrity was assessed by 1% agarose gel electrophoresis. The V3–V4 hypervariable regions of the 16S rRNA gene were amplified using the extracted genomic DNA as a template. The PCR reaction mixture had a total volume of 25 μL, consisting of 2.5 μL of 10× Buffer, 2 μL of dNTPs (2.5 mM), 0.5 μL each of forward and reverse primers (10 μM), 0.25 μL of Taq DNA polymerase (5 U/μL), and 1 μL of DNA template. The reaction conditions were as follows: initial denaturation at 94 °C for 5 min; 30 cycles of denaturation at 94 °C for 30 s, annealing at 54 °C for 30 s, and extension at 72 °C for 45 s; followed by a final extension at 72 °C for 10 min. The PCR products were verified by 2% agarose gel electrophoresis and then purified and recovered. Paired-end sequencing (2 × 250 bp) was performed on the Illumina NovaSeq 6000 platform. To minimize potential exogenous contamination, strict precautions were implemented throughout the workflow, including the use of sterile individually wrapped flocked swabs and tubes, wearing of sterile gloves and surgical masks by operators, separation of DNA extraction and PCR setup in dedicated laminar flow hood areas, and exclusive use of filter tips for all pipetting steps.

### Sequencing data processing and bioinformatics analysis

Raw sequencing data were processed with QIIME2. The main steps included: (1) quality control, denoising, read merging, and chimera removal of raw sequences using the DADA2 plugin to obtain amplicon sequence variants (ASVs); (2) clustering into operational taxonomic units (OTUs) at 97% similarity; (3) taxonomic assignment using the SILVA v138 database; (4) rarefying the OTU table to an even sequencing depth for downstream analyses.

Alpha diversity was assessed using the Shannon index and Chao1 index, and differences between groups were compared using the Mann–Whitney U test. Beta diversity was evaluated by principal component analysis (PCA) based on the Bray–Curtis distance, and differences in community structure between groups were assessed using permutational multivariate analysis of variance. Taxonomic composition at the phylum and genus levels was visualized via stacked bar charts depicting relative abundances.

### Differential species analysis

LEfSe analysis was employed to identify microbial biomarkers with significant differences between groups, with screening criteria set as a linear discriminant analysis (LDA) score >2.0 and P < 0.05. ANCOM-BC and MaAsLin2 were further utilized to corroborate the differentially abundant species. Relative abundances of differentially abundant bacterial genera were visualized in heatmaps following Z-score normalization. A microbial co-occurrence network was constructed based on Spearman’s rank correlation coefficient (|ρ| > 0.5, P < 0.05), and network visualization was performed using Gephi software.

### Construction of diagnostic models

To avoid data leakage, all genera were directly input into the LASSO regression model without any prior feature filtering. Feature selection was performed entirely within each fold of the 10-fold cross-validation. Under the optimal regularization parameter λ.min, three genera with non-zero coefficients (Fibrobacter, Ruminococcaceae_UCG-002, and Staphylococcus) were selected and incorporated into the final diagnostic model. Model performance was evaluated via 10-fold cross-validation, and model stability was further validated using 100 Bootstrap resampling iterations (the Bootstrap procedure also adopted an internal feature selection strategy, wherein features were independently selected within the training set and evaluated on the validation set in each iteration).

### Screening of recurrence-related candidate biomarkers

Given the limited number of recurrence events (n = 10), multivariable modeling was statistically infeasible. Therefore, rather than constructing a multivariable recurrence prediction model, a conservative exploratory univariate screening strategy was adopted to identify candidate microbial biomarkers associated with postoperative recurrence. To avoid overfitting and data leakage, Bootstrap validation with internal feature selection (300 iterations) was employed: in each iteration, the univariate AUC of each genus was calculated within the training set, the genus with the highest AUC in that iteration was selected, and its performance was evaluated on the validation set. The selection frequency of each genus across iterations served as a measure of its stability. All recurrence-related results were explicitly designated as exploratory findings.

### Integration of clinical parameters

Clinical data of the participants were collected, including age, sex, smoking history, allergic rhinitis, obesity, peripheral blood eosinophil ratio, Lund-Mackay CT score, Lund-Kennedy endoscopic score, VAS symptom score, and comorbid asthma. Spearman’s rank correlation analysis was performed to assess the correlations between the abundance of key genera and clinical parameters. The eosinophil-related indicator used in this study was the peripheral blood eosinophil ratio; no histopathological evaluation of eosinophilic infiltration in polyp tissues was performed.

### Statistical analysis

Continuous variables were summarized as mean ± standard deviation or median (interquartile range), and group comparisons were conducted using the Mann–Whitney U test (for two groups) or the Kruskal–Wallis test (for multiple groups). Categorical variables were presented as frequencies (percentages), and group comparisons were conducted using the chi-square test or Fisher’s exact test. Associations were assessed using Spearman’s rank correlation coefficient. Feature importance for each bacterial genus was evaluated using the random forest algorithm, based on the mean decrease in the Gini index. All statistical analyses were conducted using R software, and a two-sided *P* value < 0.05 was considered statistically significant.

## Results

### Compositional alterations of the nasopharyngeal microbiota in patients with chronic rhinosinusitis with nasal polyps

To characterize the nasopharyngeal microbiota in patients with CRSwNP, nasopharyngeal swab samples from 32 patients with CRSwNP and 11 patients with nasal septum deviation (control group) were collected and analyzed by 16S rRNA gene sequencing (**Table 1**). The results revealed significant compositional alterations in the nasopharyngeal microbiota between the CRSwNP group and the control group (**Supplementary Figure 1A–C**). We compared the phylum-level composition (relative abundance ≥1%) of the nasopharyngeal microbiota between the two groups. Among the dominant phyla, Proteobacteria, Firmicutes, and Actinobacteria were significantly enriched in the control group, whereas Bacteroidetes was significantly enriched in the CRS group (**Figures 1A, B**). Regarding rare phyla, Acidobacteria, Spirochaetes, Tenericutes, Chloroflexi, and Fibrobacteres were also markedly enriched in the CRSwNP group (**Figure 1C**).

**Table 1:** Comparison of clinical baseline characteristics between patients with CRSwNP and controls.

Characteristics	Control	CRSwNP	pvalue
n	11	32	
Gender, n (%)			0.335
Female	3 (7%)	14 (32.6%)	
Male	8 (18.6%)	18 (41.9%)	
Age, mean ± sd	39.455 ± 8.6529	47.625 ± 11.03	0.032
Smoking, n (%)			0.190
No	8 (18.6%)	16 (37.2%)	
Yes	3 (7%)	16 (37.2%)	
Allergic rhinitis, n (%)			0.642
No	6 (14%)	20 (46.5%)	
Yes	5 (11.6%)	12 (27.9%)	
Obesity, n (%)			0.539
No	8 (18.6%)	20 (46.5%)	
Yes	3 (7%)	12 (27.9%)	
Eosinophil Ratio, mean ± sd	2.7868 ± 1.0408	5.2313 ± 1.3768	<0.001
Lund-Mackay Score, mean ± sd	0.63636 ± 0.6742	9.625 ± 3.6874	<0.001
Lund-Kennedy Score, mean ± sd	0.54545 ± 0.68755	6.5938 ± 3.3874	<0.001
VAS Score, mean ± sd	2.2727 ± 1.2721	5.1562 ± 1.9856	<0.001

CRSwNP, chronic rhinosinusitis with nasal polyps; VAS, Visual Analog Scale; n, sample size. Percentages are based on group-specific sample sizes. P < 0.05 was considered statistically significant.

**Figure 1:**
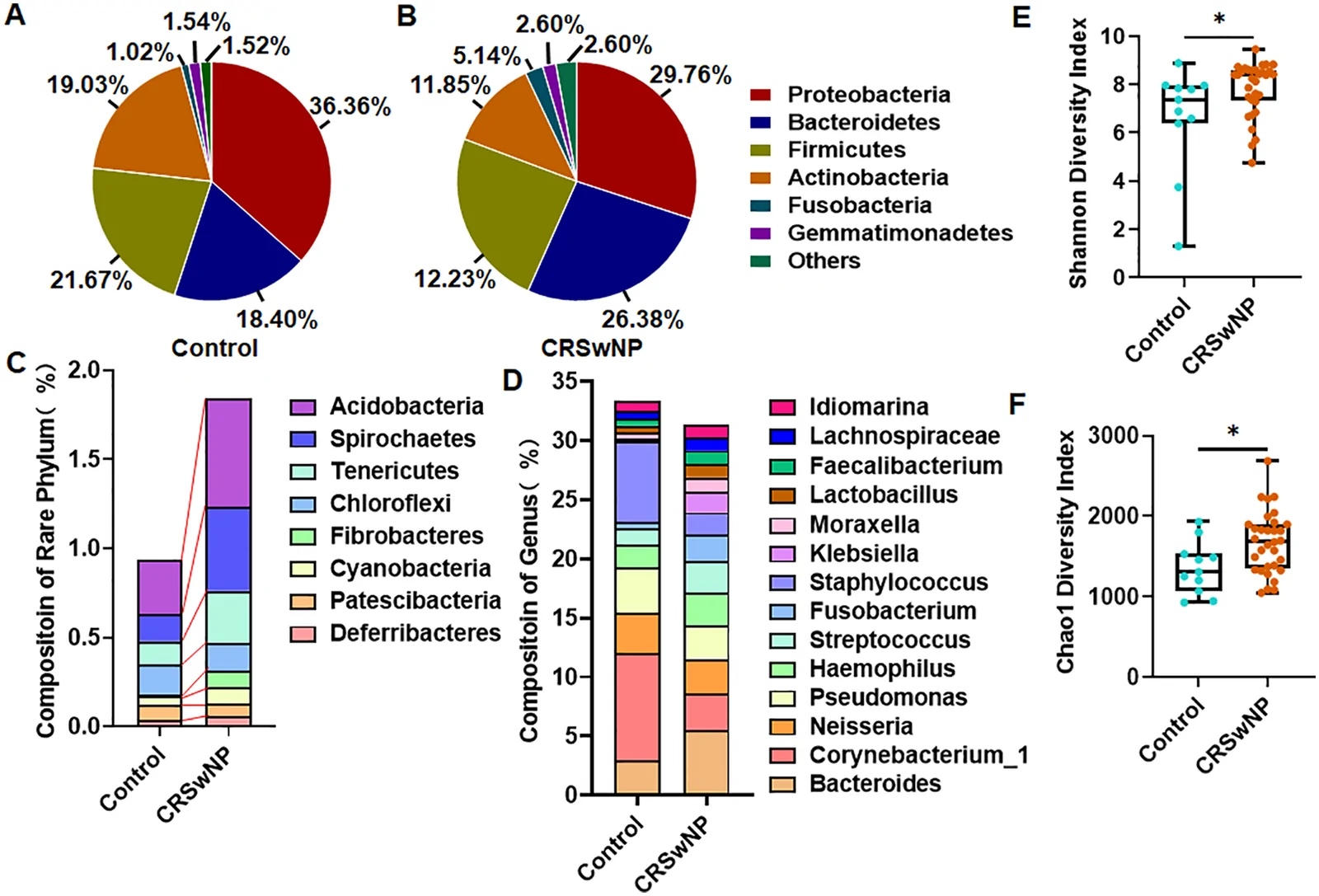
Comparison of nasopharyngeal microbiota composition and diversity between patients with CRSwNP and controls. **(A)** The microbial composition at phylum level in CRSwNP group; **(B)** The microbial composition at phylum level in control groups, Phylum with relative population above 1% was denoted as abundant, while “Other” was denoted as the sum of rare phyla with relative population less than 1%; **(C)** Rare phylum composition in CRSwNP and control group; **(D)** Genus composition in CRSwNP and control group; **(E)** Alpha diversity for Shannon index at genus level; **(F)** Alpha diversity for Chao1 index at genus level.

At the genus level, significant compositional differences in the microbiota were also observed between the two groups (**Figure 1D**). Further analysis of species diversity revealed significantly higher microbial diversity in the CRS group nasopharynx compared with the control group. Specifically, both the Shannon index and Chao1 index were significantly elevated in the CRS group relative to the control group (**Figures 1E, F**).

To identify microbial biomarkers with significant differences between the two groups, Linear Discriminant Analysis Effect Size (LEfSe) a analysis was performed to identify microbial biomarkers exhibiting significant differential abundance between the two groups (**Figure 2A**). A heatmap further displayed the abundance distribution patterns of differentially abundant genera in samples from both groups (**Figure 2B**). Subsequently, Spearman’s rank correlation coefficient was used to analyze correlations among the differential genera, with node sizes in the correlation heatmap reflecting the strength of the correlation coefficients (**Figure 2C**). Finally, a nasopharyngeal microbial co-occurrence network was constructed based on core bacterial genera, depicting the microbial interaction patterns in the control group and the CRS group, respectively (**Figures 2D, E**). The results indicated that the microbial network structure in the CRS group exhibited greater complexity than that of the control group, suggesting a remodeling of microbial interactions within the nasopharyngeal community under disease conditions.

**Figure 2:**
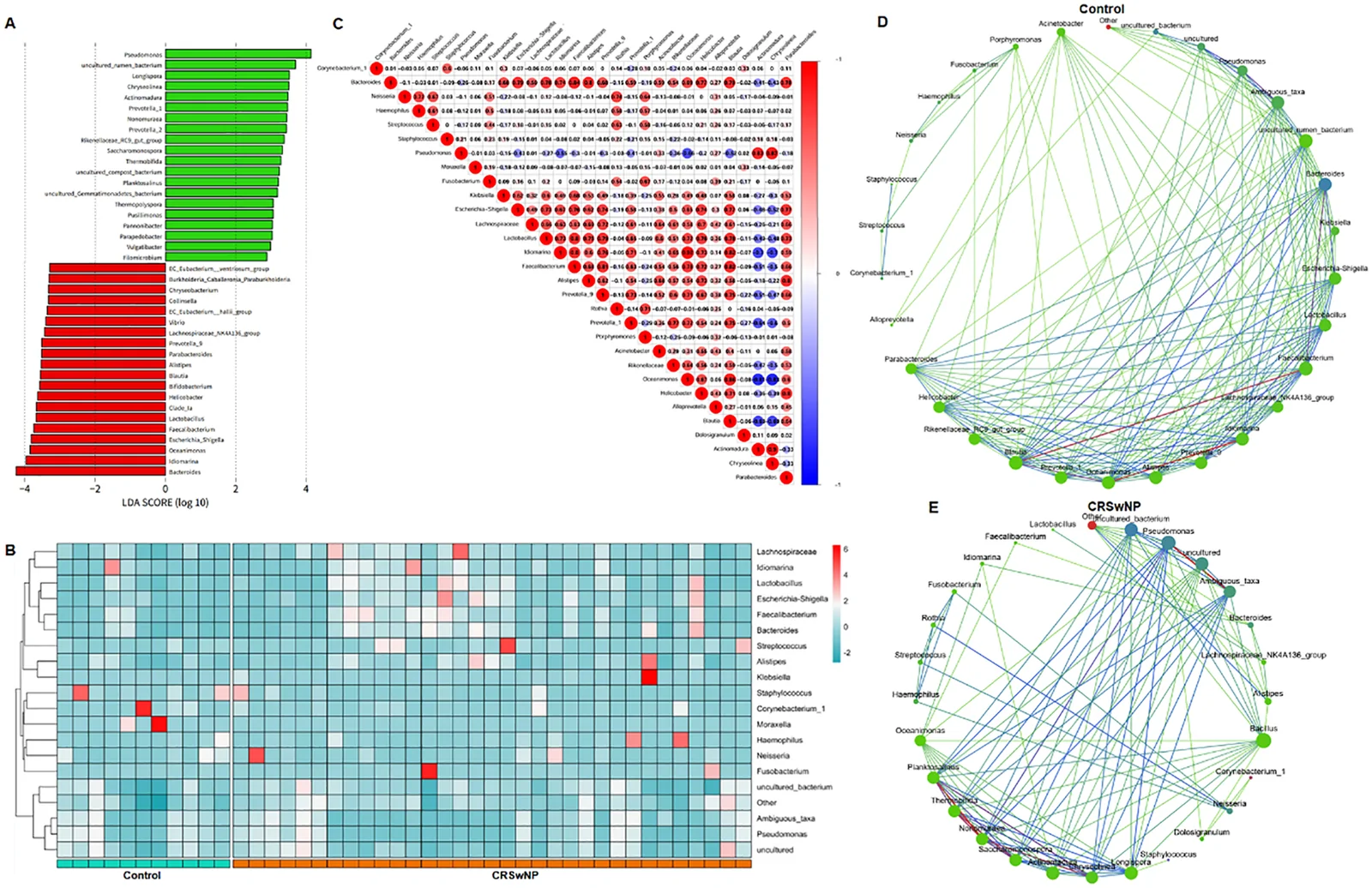
Differential analysis and co-occurrence network of nasopharyngeal microbiota between patients with CRSwNP and controls. **(A)** LDA score plot from LEfSe analysis showing the effect size of differentially abundant taxa between the two group (LDA score > 2.0, P < 0.05); **(B)** Heatmap of differentially abundant genera showing the top 30 genera with significantly different relative abundances between the two group; **(C)** Correlation heatmap of differentially abundant genera based on Spearman’s rank correlation coefficient, where node size represents the strength of the correlation coefficient; **(D)** Co-occurrence network of nasopharyngeal microbiota in the control group; **(E)** Co-occurrence network of nasopharyngeal microbiota in the CRS group. Green nodes indicate genera enriched in the CRS group, and red nodes indicate genera enriched in the control group. Node size represents the mean relative abundance, and edge width represents the strength of correlation (Spearman’s |ρ| > 0.5, P < 0.05).

### Construction and evaluation of a diagnostic model for chronic rhinosinusitis with nasal polyps based on microbial biomarkers

To avoid data leakage, all genera were directly input into the LASSO regression model, with feature selection performed entirely within each fold of the 10-fold cross-validation. Under the optimal λ.min, three genera (*Fibrobacter, Ruminococcaceae_UCG-002, and Staphylococcus*) retained non-zero coefficients and were incorporated into the final diagnostic model (**Figure 3A****;** **Supplementary Figures 2A–C**). This three-genus combined model achieved an AUC of 0.943 in the 10-fold cross-validation (**Figure 3B**). The mean AUC from the 100 Bootstrap iterations with internal feature selection was 0.890 (**Figures 3C, D**), further supporting the stability of the model. The calibration curve demonstrated good agreement between predicted probabilities and observed outcomes (**Figure 3E**). Decision curve analysis indicated that the model provided clinical net benefit across a wide range of threshold probabilities (**Figure 3F****;** **Supplementary Figure 2D**).

**Figure 3:**
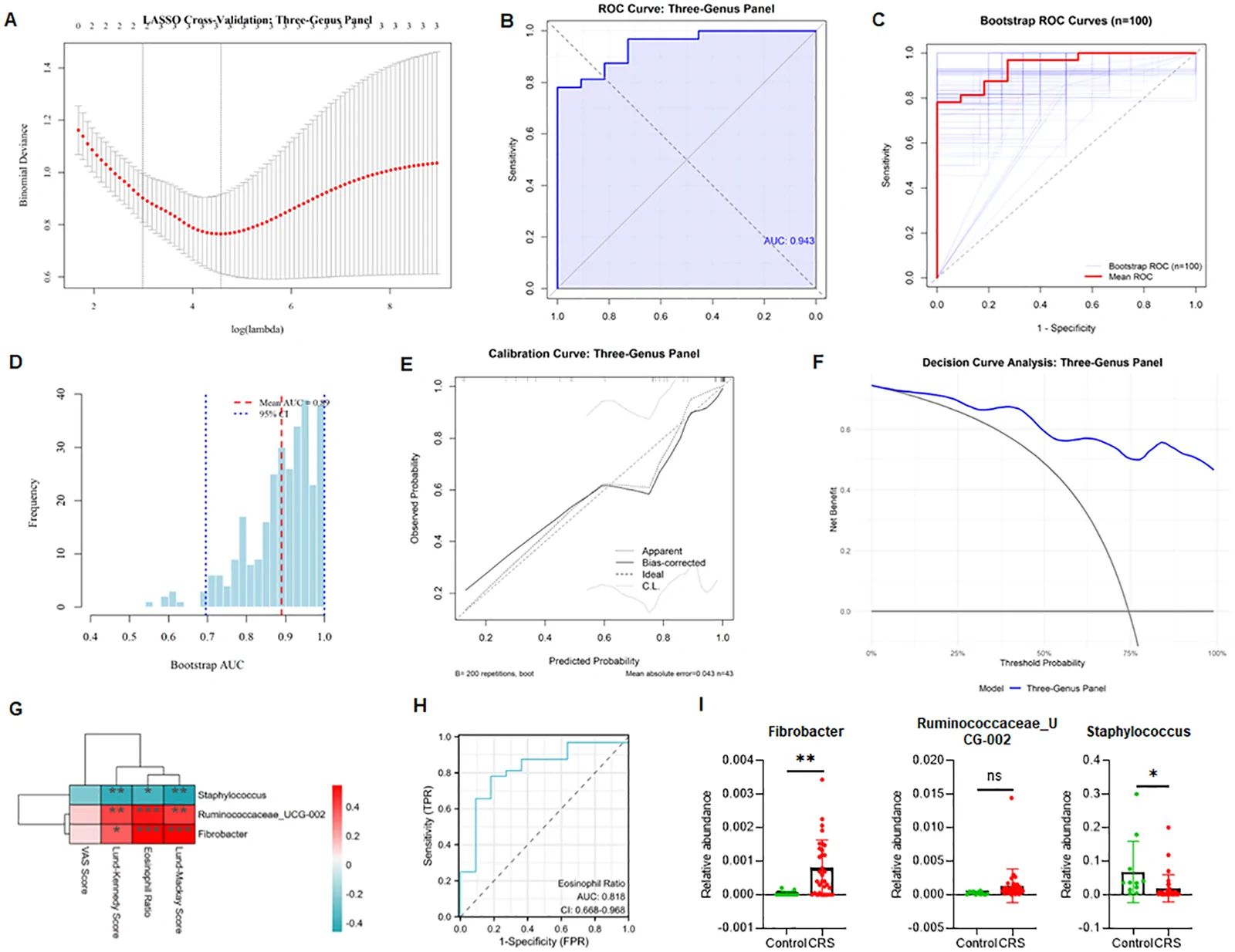
Screening and validation of microbial biomarkers for discriminating CRSwNP. **(A)** LASSO regression model for selecting optimal predictive features via binomial deviance minimization; **(B)** ROC curve of the three-gene combined diagnostic model; **(C)** ROC curves from 100 Bootstrap resampling iterations and the mean ROC curve; **(D)** Histogram of AUC distribution with 95% confidence interval for the three-gene diagnostic model based on 100 Bootstrap resampling iterations; **(E)** Calibration curve of the three-gene diagnostic model, showing agreement between predicted probabilities and observed outcomes, **(F)** Decision curve analysis (DCA) of the three-gene diagnostic model for evaluating clinical net benefit; **(G)** Correlation heatmap of microbial genus-level abundances with clinical scores (Lund–Mackay score, Lund–Kennedy score, VAS score, and peripheral blood eosinophil ratio); **(H)** ROC curve of the clinical model based on peripheral blood eosinophil ratio; **(I)** Comparison of relative abundance differences of the specific genera included in the three-gene model between the control group and the CRS group.

Spearman correlation analysis revealed that the abundances of Fibrobacter and Ruminococcaceae_UCG-002 were positively correlated with Lund-Mackay scores, Lund-Kennedy scores, and peripheral blood eosinophil ratios, while the abundance of Staphylococcus was also positively correlated with Lund-Mackay scores, Lund-Kennedy scores, and peripheral blood eosinophil ratios. No significant correlations were observed between any of the three genera and VAS scores (**Figure 3G**). ROC curve comparison demonstrated that the three-genus combined model outperformed peripheral blood eosinophil ratio, a single clinical indicator (**Figure 3H**). Finally, a combination of boxplots for the key diagnostic genera intuitively displayed the abundance differences of each key genus between the control group and the CRSwNP group, further validating the potential of these genera as diagnostic biomarkers (**Figure 3I**).

### Differential analysis of nasopharyngeal microbiota between recurrent and non-recurrent chronic rhinosinusitis with nasal polyps patients

To investigate the differences in the nasopharyngeal microbiota between recurrent and non-recurrent CRSwNP patients, we analyzed 16S rRNA gene sequencing data from nasopharyngeal swab samples of the two groups (**Table 2**). The results revealed significant compositional alterations in the nasopharyngeal microbiota of recurrent CRS patients compared with non-recurrent patients (**Supplementary Figures 3A–C**). At the phylum level, marked differences in microbial composition were observed between the two groups (relative abundance ≥1%). Specifically, major phyla exhibited distinct distribution patterns between the recurrent and non-recurrent groups (**Figures 4A, B**). Among less abundant phyla, Cyanobacteria and Fibrobacteres were significantly enriched in the recurrent group, whereas Tenericutes and Spirochaetes were enriched in the non-recurrent group (**Figure 4C**). Significant compositional differences in the microbiota were also observed at the genus level between the two groups (**Figure 4D**). Further analysis of species diversity revealed a significantly higher microbial diversity in the nasopharynx of the recurrent group compared with the non-recurrent group. Specifically, both the Shannon and Chao1 indices were significantly elevated in the recurrent group relative to the non-recurrent group (**Figures 4E, F**).

**Table 2:** Comparison of clinical baseline characteristics between patients with recurrent and non-recurrent CRSwNP.

Characteristics	Non- recurrent	Recurrent	pvalue
n	22	10	
Gender, n (%)			0.124
Female	12 (37.5%)	2 (6.2%)	
Male	10 (31.2%)	8 (25%)	
Age, mean ± sd	47.5 ± 9.7675	47.9 ± 14.012	0.926
Smoking, n (%)			0.252
No	13 (40.6%)	3 (9.4%)	
Yes	9 (28.1%)	7 (21.9%)	
Allergic rhinitis, n (%)			0.018
No	17 (53.1%)	3 (9.4%)	
Yes	5 (15.6%)	7 (21.9%)	
Obesity, n (%)			0.438
No	15 (46.9%)	5 (15.6%)	
Yes	7 (21.9%)	5 (15.6%)	
Eosinophilia, n (%)			<0.001
No	19 (59.4%)	2 (6.2%)	
Yes	3 (9.4%)	8 (25%)	
History of sinus surgery, n (%)			0.407
No	17 (53.1%)	6 (18.8%)	
Yes	5 (15.6%)	4 (12.5%)	
Eosinophil Ratio, mean ± sd	4.8755 ± 1.2845	6.014 ± 1.2997	0.028
Lund-Mackay Score, mean ± sd	8.4545 ± 3.1279	11 ± 3.496	0.048
Lund-Kennedy Score, mean ± sd	5.1364 ± 2.1667	9.8 ± 3.4577	0.002
VAS Score, mean ± sd	4.4091 ± 1.7904	6.8 ± 1.3166	<0.001

CRSwNP, chronic rhinosinusitis with nasal polyps; VAS, Visual Analog Scale; n, sample size. Percentages are based on the total sample size (N = 32). P < 0.05 was considered statistically significant.

**Figure 4:**
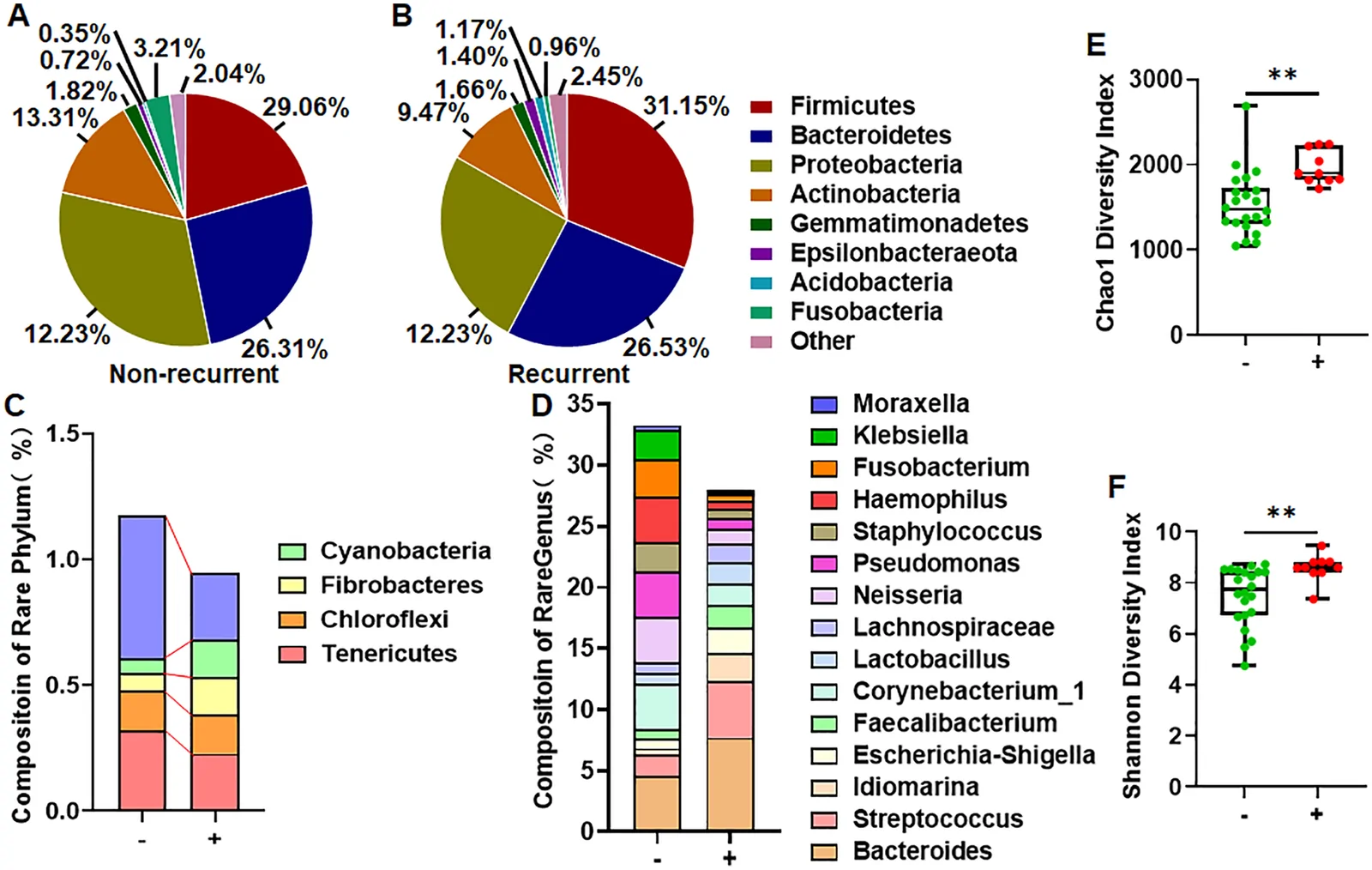
Comparison of nasopharyngeal microbiota composition and diversity between patients with recurrent and non-recurrent CRSwNP. **(A)** The microbial composition at phylum level in non-recurrent group; **(B)** The microbial composition at phylum level in recurrent group, Phylum with relative population above 1% was denoted as abundant, while “Other” was denoted as the sum of rare phyla with relative population less than 1%; **(C)** Rare phylum composition in recurrent and non-recurrent group; **(D)** Genus composition in recurrent and non-recurrent group; **(E)** Alpha diversity for Shannon index at genus level; **(F)** Alpha diversity for Chao1 index at genus level. -: non-recurrent group; +: recurrent group.

LEfSe analysis was performed to identify microbial biomarkers exhibiting significant differential abundance between the two groups. The LDA score plot depicted the effect sizes of differentially abundant taxa, revealing that multiple bacterial genera differed significantly in abundance between the two groups (**Supplementary Figure 4A**). A heatmap further displayed the abundance distribution patterns of differentially abundant genera in samples from both groups (**Supplementary Figure 4B**). Finally, a nasopharyngeal microbial co-occurrence network was constructed based on core bacterial genera, elucidating the microbial interaction patterns in the non-recurrent and recurrent groups, respectively (**Supplementary Figures 4C, D**). The results indicated that the microbial network structure in the recurrent group was markedly remodeled compared with that in the non-recurrent group, suggesting an alteration in microbial interactions within the nasopharyngeal community under disease recurrence conditions.

### Construction and evaluation of a recurrence prediction model for chronic rhinosinusitis with nasal polyps based on microbial biomarkers

Given the limited number of recurrence events (n=10), this study performed exploratory univariate screening to identify candidate genera associated with postoperative recurrence. Among the 32 CRSwNP patients, univariate logistic regression analysis identified *Parabacteroides* (AUC = 0.955) and *Helicobacter* (AUC = 0.977) as the two genera most strongly associated with postoperative recurrence (**Figures 5A, B**). ROC curve comparison analysis demonstrated that both *Helicobacter* and *Parabacteroides* exhibited superior discriminatory performance compared to single clinical indicators, including peripheral blood eosinophil ratio, Lund-Mackay score, Lund-Kennedy score, and VAS score (**Supplementary Figures 5A–D**). To evaluate the stability of candidate biomarkers, Bootstrap validation with internal feature selection (300 iterations) was employed (**Figure 5C**). *Parabacteroides* was the most frequently selected genus (27.3%), followed by *Helicobacter* (24.0%), suggesting that these two genera exhibited favorable stability among recurrence-related biomarkers (**Figure 5C**). The correlation network diagram illustrated the co-occurrence patterns among candidate genera (**Figure 5D**), and heatmap clustering analysis revealed a partially separated clustering pattern between the recurrent and non-recurrent groups (**Figure 5E**). Boxplot analysis further demonstrated that the relative abundances of both *Parabacteroides* and *Helicobacter* were significantly higher in the recurrent group than in the non-recurrent group (**Figure 5F**), supporting the biological plausibility of these two genera as recurrence candidate biomarkers. The correlation heatmap between core microbial abundances and clinical scores showed that the abundances of *Parabacteroides* and *Helicobacter* were significantly positively correlated with clinical parameters including Lund-Kennedy scores and VAS scores (**Figure 5G**). Importantly, all recurrence-related findings presented above are exploratory hypothesis-generating discoveries rather than confirmatory conclusions. Due to the limited sample size, these findings cannot serve as a basis for clinical decision-making and require validation in an independent large-scale cohort before their clinical application can be considered.

**Figure 5:**
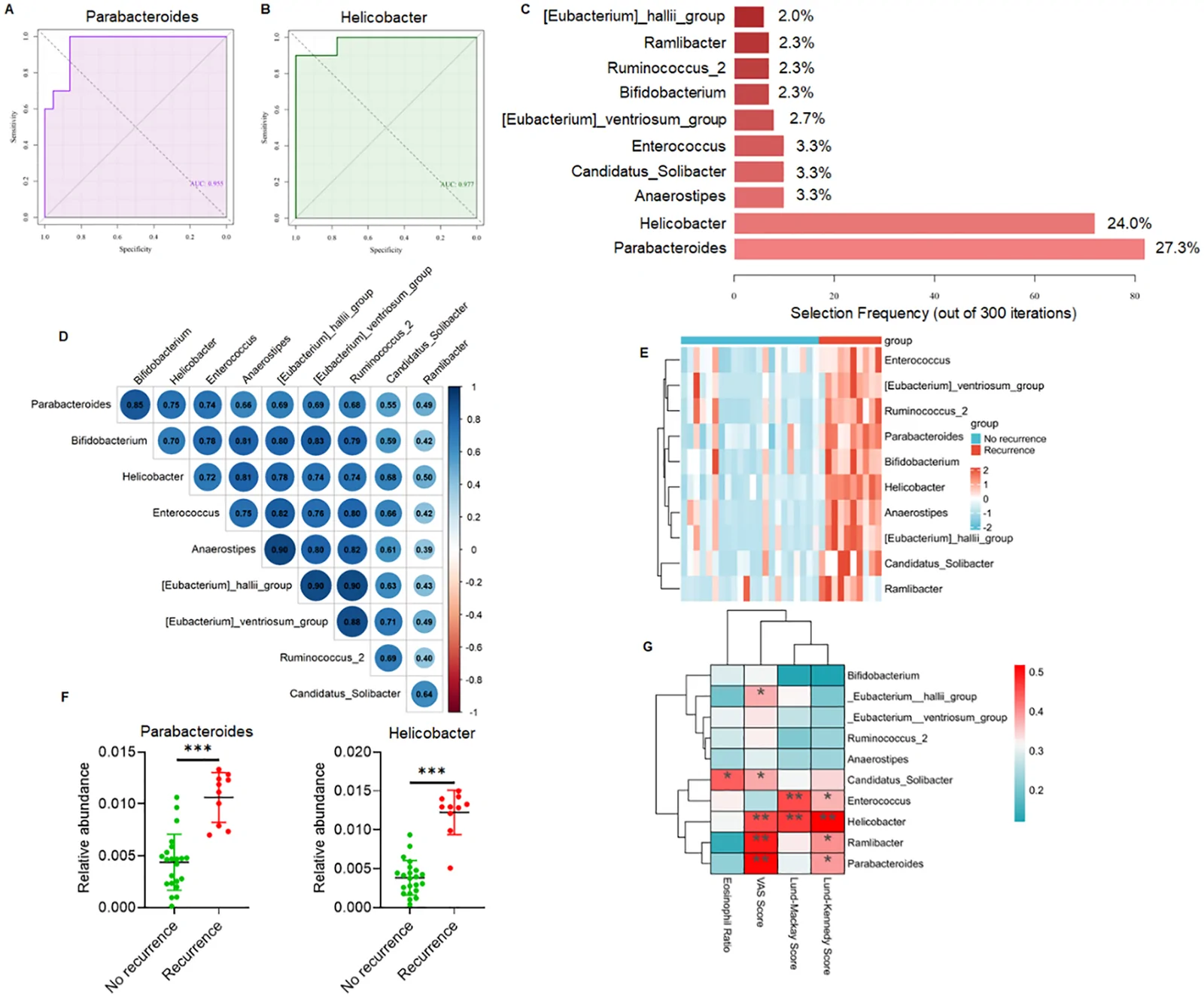
Core microbiota associated with CRSwNP recurrence and clinical correlation analysis. **(A, B)** ROC curves and AUC values of *Parabacteroides* and *Helicobacter* alone for predicting recurrence; **(C)** Key genus-level microorganisms highly associated with CRSwNP recurrence selected based on selection frequency across 300 iterations; **(D)** Correlation network diagram among the selected key microorganisms; **(E)** Heatmap clustering analysis of core microbial abundances between the recurrence group and the non-recurrence group; **(F)** Comparison of relative abundance differences of *Parabacteroides* and *Helicobacter* between the recurrence and non-recurrence groups; **(G)** Correlation heatmap of core microbial abundances with various clinical scores. *P < 0.05, **P < 0.01, *P < 0.001.

### Clinical relevance of microbial biomarkers and subgroup analyses

We employed the random forest algorithm to rank the feature importance of the top 10 genera, and the resulting bar plot illustrated the relative contribution of each genus in discriminating recurrent from non-recurrent CRSwNP patients, while also distinguishing the importance of microbial features from that of clinical features (**Figure 6A**). Further Spearman correlation analysis between differentially abundant genera and clinical indicators revealed that key genera were significantly correlated with eosinophil ratio, Lund–Mackay score, Lund–Kennedy score, and VAS score, suggesting that microbial alterations are strongly associated with the risk of disease recurrence (**Figure 6B**). Additionally, a panel of boxplots for the key genera clearly depicted the abundance differences of each genus between the recurrent and non-recurrent groups (**Figure 6C**).

**Figure 6:**
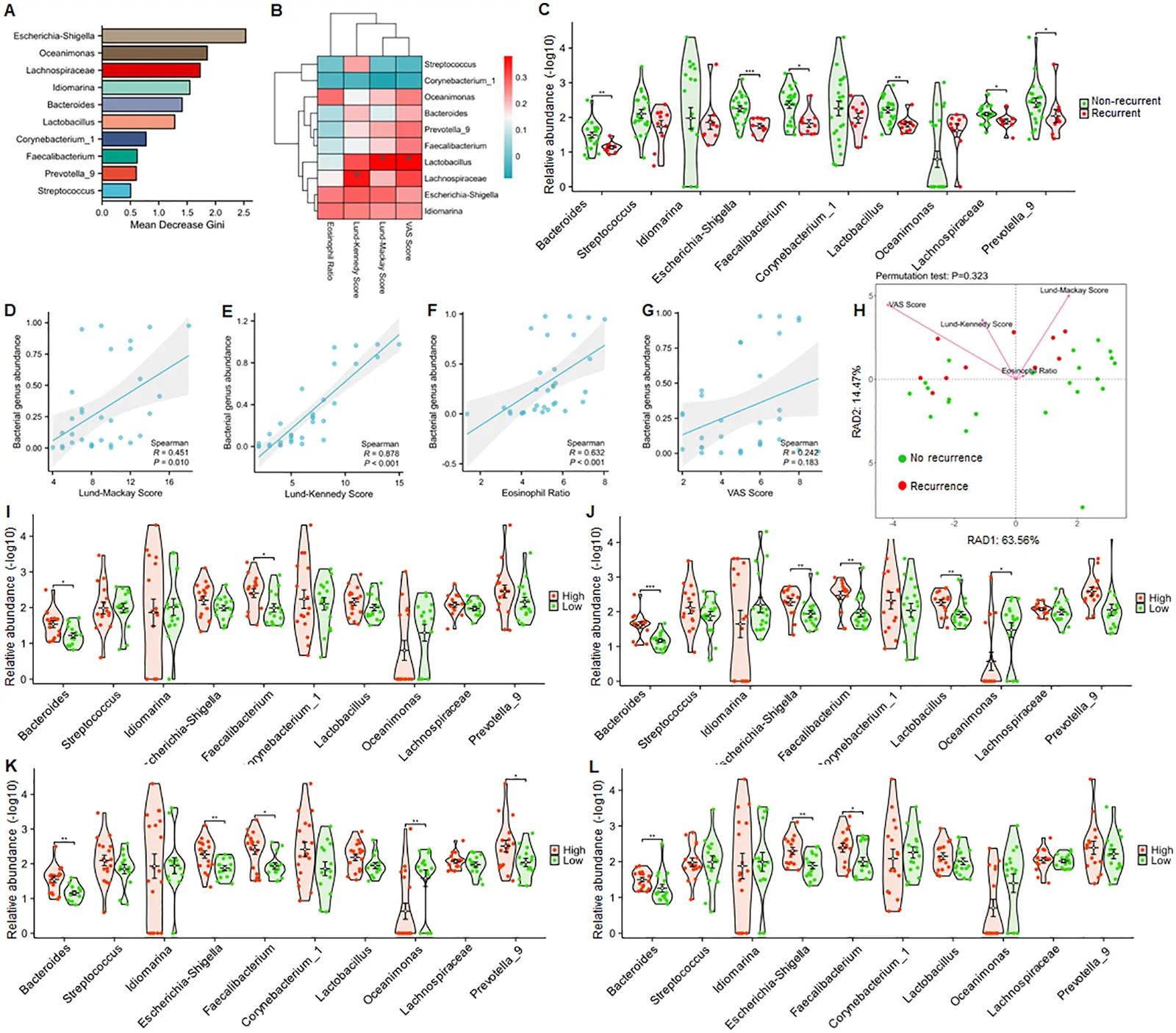
Clinical relevance and subgroup analysis of the microbial marker composite index. **(A)** Spearman correlation heatmap between differentially abundant genera and clinical parameters; **(B)** Random forest feature importance bar plot showing the importance ranking of the top 10 genera; **(C)** Combined boxplot panel of key diagnostic genera showing abundance differences between the control group and the CRSwNP group. **(D)** Scatter plot of the correlation between the microbial marker composite index and Lund-Kennedy score; **(E)** Scatter plot of the correlation between the microbial marker composite index and Lund-Mackay score; **(F)** Scatter plot of the correlation between the microbial marker composite index and eosinophil ratio; **(G)** Scatter plot of the correlation between the microbial marker composite index and VAS score; **(H)** PCA biplot with clinical environmental vectors showing the relationship between clinical factors and microbial community structure, where arrow directions indicate the direction and strength of environmental factors; **(I)** Boxplots of key genus abundances stratified by Lund-Mackay score; **(J)** Boxplots of key genus abundances stratified by Lund-Kennedy score; **(K)** Boxplots of key genus abundances stratified by eosinophil ratio; **(L)** Boxplots of key genus abundances stratified by VAS score. *P < 0.05, **P < 0.01, *P < 0.001.

To further validate the clinical relevance of the microbial biomarkers, we constructed a composite microbial biomarker index by weighting multiple key bacterial genera based on their logistic regression coefficients and examined its correlation with indicators of disease severity. Scatter plots with regression lines revealed a significant positive correlation between the composite index and both the Lund–Kennedy score (**Figure 6D**) and the Lund–Mackay score (**Figure 6E**), suggesting that a higher composite index is associated with more severe pathological changes assessed by imaging and endoscopy. Additionally, the composite index was significantly positively correlated with eosinophil ratio (**Figure 6F**) and the VAS score (**Figure 6G**), suggesting a close association between this index and the patient’s systemic inflammatory status and the severity of subjective symptoms.

To evaluate the robustness of the microbial biomarker model across different clinical subgroups, we generated stratified ROC curves to assess its diagnostic and predictive performance in patients with or without comorbid asthma or rhinitis. The results showed that the model maintained good discriminatory ability across all subgroups (**Supplementary Figure 6**), suggesting that the performance of this microbial biomarker model remains robust regardless of asthma or rhinitis comorbidity and exhibits good generalizability.

We further employed PCA overlaid with vectors representing clinical variables to explore the relationship between clinical factors and nasopharyngeal microbial community structure. The results indicated that environmental factors explained a certain proportion of the variance in the microbial community structure. Specifically, community variation in the non-recurrent group was primarily driven by the VAS score, suggesting that subjective symptoms play a dominant role in shaping the microbial community in this group. In contrast, community variation in the recurrent group was more dependent on the Lund–Mackay score, indicating that objective imaging indicators are more closely associated with the microbial community structure under recurrent disease conditions (**Figure 6H**).

To further elucidate the abundance patterns of key bacterial genera across different clinical strata, we performed multifactorial stratified analyses. After stratification by Lund–Mackay score, boxplots showed significant differences in the abundance of key genera between the high-score and low-score groups (**Figure 6I**). Significant differences were also observed after stratification by Lund–Kennedy score (**Figure 6J**). Analyses stratified by eosinophil percentage (**Figure 6K**) and VAS score (**Figure 6L**) further confirmed that the abundance of key genera was significantly higher in the high-score groups across all clinical indicators, indicating that these microbial biomarkers reflect multiple dimensions of disease severity.

In conclusion, nasopharyngeal microbial biomarkers are closely correlated with multiple clinical indicators of CRS disease severity and maintain stable predictive performance across different clinical subgroups. Furthermore, they exhibit distinct drivers of microbial community structure depending on recurrence status, suggesting that these microbial biomarkers have broad clinical applicability.

## Discussion

In this study, we systematically characterized the nasopharyngeal microbiota composition of CRSwNP patients via 16S rRNA gene sequencing, constructed a diagnostic model based on LASSO with internal feature selection within cross-validation, and exploratorily identified candidate microbial biomarkers associated with postoperative recurrence. Our findings revealed microbial biomarkers with potential diagnostic value, providing new insights into the non-invasive diagnosis of CRSwNP. Our results revealed significant differences in nasopharyngeal microbial composition between CRSwNP patients and controls at both the phylum and genus levels. At the phylum level, *Proteobacteria*, *Firmicutes*, and *Actinobacteria* were significantly enriched in the control group, whereas *Bacteroidetes* showed significant enrichment in the CRSwNP group. These findings are largely consistent with those of previous studies. Previous research has established that the nasal microbiota of healthy individuals is typically dominated by *Actinobacteria* and *Firmicutes*, whereas patients with CRS exhibit increased relative abundances of *Proteobacteria* and *Bacteroidetes* (Paramasivan et al., 2020). At the genus level, this study identified significant differences in multiple bacterial genera between the two groups, characterized by a significant enrichment of *Staphylococcus* in CRSwNP patients and a higher abundance of *Corynebacterium* in the control group. This pattern is consistent with established paradigms regarding the upper respiratory microbiome, in which *Corynebacterium* and *Dolosigranulum* dominate under healthy conditions, whereas potentially pathogenic bacteria such as *Staphylococcus* proliferate under disease conditions (Paramasivan et al., 2020; Connell et al., 2024; Ryser et al., 2025). *Corynebacterium* is posited to exert a protective effect by producing antimicrobial peptides and competitively inhibiting pathogen colonization to maintain mucosal homeostasis (Kim et al., 2020; Paramasivan et al., 2020). In contrast, *Staphylococcus*, particularly *Staphylococcus aureus*, is known to produce superantigens that activate local immune responses, promote Th2-driven inflammation, and facilitate polyp formation (Connell et al., 2024). The findings of this study further substantiate the pathogenic role of *Staphylococcus* in the pathogenesis of CRSwNP and the protective role of *Corynebacterium*.

It is important to emphasize that our sampling targeted the nasopharynx, rather than the sinus mucosa or the inferior/middle turbinates. The nasopharynx serves as a critical ecological niche and a “microbial hub” connecting the upper and lower respiratory tracts. The nasopharyngeal microbiota may reflect both local sinonasal microbial dynamics and systemic immune-microbial interactions. While sinus mucosal biopsy or turbinate swabbing may provide site-specific information, nasopharyngeal swabbing offers a minimally invasive, clinically feasible approach suitable for outpatient settings.

Notably, this study found that both the Shannon index and the Chao1 index were significantly higher in CRSwNP patients than in the control group. These findings contrast with those of several prior studies. Preceding studies have reported reduced nasal microbial diversity in CRSwNP patients compared with healthy controls, suggesting that the microbial community shifts toward a “simplified” state dominated by a single overgrowing genus under disease conditions (Ryser et al., 2025). However, some studies have observed the opposite trend, specifically, an increase in microbial diversity among CRSwNP patients (Zhao et al., 2022). Possible factors contributing to this discrepancy include differences in sampling site (nasopharynx vs. nasal cavity vs. sinuses), disease severity, medication history (antibiotic or corticosteroid use), and sequencing/analysis methodologies. One speculative explanation is that the elevated diversity may reflect an ecologically unstable state, i.e., “pseudo-diversity,” although this hypothesis warrants further investigation. Our findings suggest that the nasopharynx, acting as a “hub” of the upper respiratory microbial community, may exhibit microbial diversity patterns distinct from those of the nasal or sinus mucosa; this hypothesis warrants further validation.

At the genus level, this study identified significant differences in multiple bacterial genera between the two groups. However, for low-abundance genera such as *Fusobacterium*, which were detected at very low relative abundances, we urge caution in interpretation, as these taxa are particularly susceptible to contamination from oral or environmental sources. The findings for these genera should therefore be considered exploratory and hypothesis-generating rather than definitive. The most robust findings in our study are those for high-abundance genera, which have been extensively characterized in prior studies and align with established paradigms in upper respiratory microbiome research.

The three-genus combined diagnostic panel (*Fibrobacter, Ruminococcaceae_UCG-002, and Staphylococcus*) constructed in this study demonstrated favorable discriminatory ability in cross-validation. Among these, *Fibrobacter* emerged as the most important discriminating genus and is of particular interest, given that it has been rarely reported in studies of upper respiratory tract diseases. The three-genus combined model outperformed traditional clinical indicators, including peripheral blood eosinophil ratio and radiological scores, suggesting that nasopharyngeal microbiota composition may provide supplementary information to routine clinical assessment. Decision curve analysis further supported its potential clinical utility.

From a translational perspective, the microbial biomarkers identified in this study offer a potential foundation for future diagnostic assay development, though we emphasize that this remains a long-term vision and is not currently a clinically available product. A feasible assay design would involve nasopharyngeal swab collection, followed by DNA extraction using commercial kits and multiplex or simplified qPCR targeting key discriminatory genera such as *Staphylococcus* and *Corynebacterium*. A scoring algorithm based on Ct values would then generate a diagnostic probability, with an estimated total turnaround time of 4–6 hours. However, we underscore that extensive analytical and clinical validation, along with regulatory approval, would be required prior to clinical implementation. Our current findings thus provide the biological rationale and candidate markers for such development, but the test itself remains a future research direction rather than an immediately available tool.

The recurrence prediction results of this study should be interpreted with caution. *Helicobacter* and *Parabacteroides* were consistently selected in the Bootstrap internal feature selection procedure, with selection frequencies of 24.0% and 27.3%, respectively, suggesting that these two genera merit further investigation as candidate biomarkers for recurrence. Boxplot analysis further confirmed that both genera were significantly enriched in the recurrent group, supporting their biological plausibility. The observed associations of *Helicobacter* and *Parabacteroides* with recurrence should be interpreted in the context of the small sample size: the high AUC values may partially reflect random fluctuations rather than true clinical utility. These findings are currently exploratory hypothesis-generating results and require independent validation in larger cohorts. Notably, this study found that microbial diversity was significantly higher in the recurrent group than in the non-recurrent group. This observation is consistent with the findings from the diagnostic phase of the study, suggesting that higher microbial diversity may be associated with disease activity and recurrence risk. It has been proposed that increased microbial diversity may reflect microbial community instability or exacerbated ecological dysbiosis rather than a marker of ecological health (Daybog and Kolodny, 2023). In pathological states, opportunistic pathogens previously suppressed by dominant commensal bacteria may proliferate extensively, leading to community structural disruption and a “pseudo-increase” in diversity (Annavajhala et al., 2019; Spragge et al., 2023). This hypothesis requires further validation through longitudinal follow-up data and functional analyses.

In this study, Spearman correlation analysis revealed that the composite microbial biomarker index showed a significant positive correlation with the Lund–Kennedy score, Lund–Mackay score, eosinophil ratio, and VAS score. These findings suggest that the extent of microbial community alteration is closely associated with the radiological severity of the disease, the severity of endoscopic lesions, systemic inflammatory status, and patients’ subjective symptoms. From a pathophysiological perspective, the overgrowth of specific bacterial genera may drive local inflammatory responses by activating Toll-like receptor signaling pathways and promoting the release of pro-inflammatory cytokines, thereby exacerbating tissue damage and polyp formation (Czaj et al., 2026). Furthermore, eosinophils, as core effector cells of Th2-type inflammation, may interact bidirectionally with microbial alterations, potentially forming a vicious cycle that ultimately contributes to disease progression and an increased risk of recurrence (Zhang et al., 2026).

Principal component analysis integrated with clinical environmental vectors further elucidated the relationship between clinical factors and microbial community structure. The results showed that community variation in the non-recurrent group was primarily driven by the VAS score, whereas that in the recurrent group was primarily associated with the Lund–Mackay score. This finding suggests that the clinical factors driving microbial community changes differ among CRSwNP patients with different disease states: subjective symptoms may play a dominant role in shaping the microbial community in non-recurrent disease, whereas objective imaging indicators are more closely associated with the microbial community in the recurrent phenotype. This observation offers novel insights into the microbiological basis of different clinical phenotypes of CRSwNP.

Several important methodological limitations should be considered when interpreting our findings. We did not include negative controls in our sequencing workflow. As a result, we could not systematically identify and remove potential contaminant sequences derived from reagents, environmental sources, or sampling procedures. This limitation is particularly relevant for low-abundance taxa (e.g., *Fusobacterium*, *Acinetobacter*, and certain rare phyla), which are more susceptible to contamination and may not genuinely represent the nasopharyngeal microbiota. While we implemented sterile procedures during sample collection and processing, the absence of negative controls prevents us from definitively attributing the presence of low-abundance genera to biological signals rather than background contamination. Consequently, our differential abundance findings, especially for low-abundance genera, should be interpreted with caution and considered exploratory. We emphasize that the clinical implications of our study are primarily derived from high-abundance, well-characterized genera such as *Staphylococcus* and *Corynebacterium*, which are less likely to be artifacts of contamination. The abundance pre-filtering strategy employed in the primary analysis of the diagnostic model, although necessary to reduce model complexity, nevertheless constituted a certain degree of data leakage and may have led to a slight overestimation of the AUC, readers should therefore exercise appropriate caution when interpreting the AUC of the diagnostic model. The relatively small sample size, particularly the limited number of recurrence events (n=10), fundamentally precluded any multivariable modeling. The strong associations observed between Helicobacter and Parabacteroides and recurrence should be interpreted in the context of this small sample size: the high AUC values may partially reflect random sampling fluctuations rather than genuine clinical utility. Consequently, we refrained entirely from constructing a multivariable recurrence prediction model, and all recurrence-related findings are exploratory hypothesis-generating results without clinical predictive capability. Furthermore, the use of peripheral blood eosinophil ratio rather than tissue eosinophil counts precluded histopathological endotyping, and given that the correlation between peripheral and tissue eosinophil levels is moderate and imperfect, our results should not be equated with histological classification. Despite these limitations, this study provides several novel insights: it confirms significant differences in nasopharyngeal microbiota composition between CRSwNP patients and controls, supporting its potential as a diagnostic biomarker, and identifies recurrence-associated candidate markers that offer targets for prospective validation. Future studies in large-scale, multicenter cohorts with the inclusion of negative controls, implementation of rigorous nested cross-validation frameworks, and incorporation of histopathological endotyping are warranted to validate the candidate biomarkers identified in this study and to elucidate their underlying biological mechanisms.

In summary, this study demonstrates that the nasopharyngeal microbiota of CRSwNP patients is significantly altered. The three-genus combined diagnostic panel consisting of *Fibrobacter*, *Ruminococcaceae_UCG-002*, and *Staphylococcus* exhibited favorable diagnostic performance and outperformed multiple traditional clinical indicators, supporting its potential as a non-invasive diagnostic biomarker. Regarding recurrence, *Helicobacter* and *Parabacteroides* were identified as stable candidate biomarkers. However, due to the limited sample size, the recurrence-related findings are exploratory hypothesis-generating results and require validation in an independent large-scale cohort before clinical application can be considered. This study provides foundational data and candidate targets for future microbiome-based diagnostic and prognostic research in CRSwNP.

## Data Availability

The data presented in the study are deposited in the NCBI Sequence Read Archive (SRA) repository, accession number PRJNA1478436.

## Supplementary material

Supplementary Figure 1 Comparative analysis of differential abundance and community structure of the nasopharyngeal microbiota between patients with CRSwNP and controls. **(A)** Principal component analysis based on genus-level relative abundance; **(B)** 3D principal component analysis plot based on genus-level relative abundance; **(C)** Volcano plot of differentially abundant microbial communities based on genus-level relative abundance.

Supplementary Figure 2 Performance comparison between the three-gene model and single-gene models. **(A–C)** ROC curves and AUC values of each single gene (*Fibrobacter, Ruminococcaceae_UCG-002*, and *Staphylococcus*) alone for diagnosing CRSwNP; **(D)** Decision curve analysis (DCA) comparing the clinical net benefit among the four models: the three-gene combined model, *Fibrobacter alone, Ruminococcaceae_UCG-002 alone*, and *Staphylococcus alone*.

Supplementary Figure 3 Comparative analysis of differential abundance and community structure of the nasopharyngeal microbiota between patients with recurrent and non-recurrent CRSwNP. **(A)** Principal component analysis based on genus-level relative abundance; **(B)** 3D principal component analysis plot based on genus-level relative abundance; **(C)** Volcano plot of differentially abundant microbial communities based on genus-level relative abundance.

Supplementary Figure 4 Differential analysis and co-occurrence network of nasopharyngeal microbiota between patients with CRSwNP and controls. **(A)** LDA score plot from LEfSe analysis showing the effect size of differentially abundant taxa between the two groups (LDA score > 2.0, P < 0.05); **(B)** Heatmap of differentially abundant genera showing the top 30 genera with significantly different relative abundances between the two groups; **(C)** Co-occurrence network of nasopharyngeal microbiota in non-recurrent group; **(D)** Co-occurrence network of nasopharyngeal microbiota in recurrent group. Green nodes indicate genera enriched in the CRS group, and red nodes indicate genera enriched in the control group. Node size represents the mean relative abundance, and edge width represents the strength of correlation (Spearman’s |ρ| > 0.5, P < 0.05).

Supplementary Figure 5 Performance of single clinical indicators for diagnosing CRSwNP. ROC curves of clinical models based on VAS score **(A)**, peripheral blood eosinophil ratio **(B)**, Lund–Kennedy score **(C)**, and Lund–Mackay score **(D)**, respectively.

Supplementary Figure 6 Stratified ROC curve analysis. Comparison of the diagnostic and predictive performance of the microbial marker model between patients with and without comorbid asthma, and between patients with and without comorbid rhinitis, demonstrating the robustness of the model across different clinical subgroups.
